# Tumor Niche Influences the Activity and Delivery of Anticancer Drugs: Pharmacology Meets Chemistry

**DOI:** 10.3390/ph18071047

**Published:** 2025-07-17

**Authors:** Mauro Ravera, Elisabetta Gabano, Stelvio Tonello, Donato Colangelo

**Affiliations:** 1 Dipartimento di Scienze e Innovazione Tecnologica, Università del Piemonte Orientale, Viale Teresa Michel 11, 15121 Alessandria, Italy; mauro.ravera@uniupo.it; 2 Dipartimento per lo Sviluppo Sostenibile e la Transizione Ecologica, Università del Piemonte Orientale, Piazza Sant’Eusebio 5, 13100 Vercelli, Italy; elisabetta.gabano@uniupo.it (E.G.); stelvio.tonello@med.uniupo.it (S.T.); 3 Dipartimento di Medicina Traslazionale, School of Medicine, Università del Piemonte Orientale, Via Solaroli 17, 28100 Novara, Italy; 4 Dipartimento di Scienze della Salute, Pharmacology, School of Medicine, Università del Piemonte Orientale, Via Solaroli 17, 28100 Novara, Italy

**Keywords:** tumor niche, tumor microenvironment chemistry, hypoxia, pH-sensitive nanocarriers, nanoparticles, precise oncology, metal-based drugs

## Abstract

Cellular and molecular characteristics of the tumor microenvironment are fundamental for the formation of niches. These structures include both cellular and matrix components and have been shown to protect and promote cancer formation and progression. The peculiarities of tumor niches have been suggested by many authors as targets with high therapeutic potential. This narrative review analyzes the chemical characteristics of the tumor microenvironment and describes experimental and clinical approaches to influence its contribution to cancer promotion and the spread of metastases. In particular, the possible chemical differences, like pH, oxygen levels, and cell composition, to be used for the design of drugs or the delivery of antiproliferative moieties for a more precise oncology approach, will be discussed. The literature proposes a vast number of molecules, but this review focuses on hypoxia-activated molecules, pH-sensitive nanocarriers, metal-based drugs, and gasotransmitters targeting selectively the tumor microenvironment as possible negative modulators of the contribution of niches to tumor promotion. The chemical peculiarities of the tumor niche are discussed for possible pharmacological developments.

## 1. Introduction

The molecular characteristics of the tumor microenvironment are fundamental for cancer progression, as well as its genotype, intratumor heterogeneity (ITH), and patient conditions [1]. Major problems in cancer chemotherapy, mainly resistance and adverse drug reactions (ADRs), are also reported for monoclonal antibodies and small molecules. These drugs represent the last frontier for precise medicine since they are directed against a peculiar target within cancer cells and have dramatically contributed to patients’ overall survival. The clinical decision for the correct use of these molecular drugs and their opportune associations with traditional chemotherapeutics is often complicated by multiple factors, such as the need for the analysis of the presence/overexpression of the target, ITH, and treatment costs [2,3,4]. 

Cancer cells acquire a progressive series of mutations and epigenetic aberrations that alter their proliferative ability and predispose them to metastases. The phenotypic characteristics of cells condition the microenvironment where they develop, which acquires a fundamental role in supporting and protecting the tumor mass [5]. Thus, the peculiarities of tumor niches (i.e., the environments in which tumors grow) have been suggested by many authors as targets with high therapeutic potential [6,7,8,9,10,11]. It was once believed that tumor growth, progression, and metastasis were intrinsically driven by the tumor. However, recent research has shown that a solid tumor is surrounded by a complex network of cells, particularly fibroblasts, macrophages, neutrophils, lymphocytes, and mesenchymal cells, which support and even promote tumor progression [12,13].

The tumor microenvironment (TME) has an extracellular matrix composed mostly of collagen and fibronectin. It forms a physical barrier that is difficult for drugs to penetrate, owing to type IV collagen capillaries, which modulate their crossing, giving the tumor a certain resistance to drug penetration. In the outermost portion, there are also cancer-associated fibroblasts (CAFs), which differ from normal fibroblasts because they present malfunctions in the regulation of gene expression at the epigenetic level. These are activated by signals released by tumor cells, such as transforming growth factor beta (TGFβ), fibroblast growth factor 2 (FGF2), and platelet derived growth factor (PDGF), and play a very important role because they secrete both growth factors, which not only support the growth and survival of malignant cells, but also act as chemotactic factors, and metalloproteases (MMPs) that allow the remodeling of the matrix [14]. CAFs have different functions, like the deposition of extracellular matrix, which creates a resistance barrier in the tumor niche, the promotion of local immunosuppression, and the epigenetic modulation of cancer cells. For this reason, there are some authors who have proposed biomimetic nanoplatforms with multiple actions, both on cancer and on cancer-associated fibroblasts [15,16].

Beyond the extracellular matrix, there is the infiltration of two particular types of cells: the former are the cells of the immune system, which are recruited by inflammatory stimuli induced by hypoxia and necrosis, caused by the low oxygen in the tumor microenvironment and which can play both a pro- and anti-tumor role; the latter are the tumor-associated endothelial cells, which are involved in the structural processes of neovascularization, due to a lower production of intercellular adhesion molecule 1 and 2 (ICAM1 and ICAM2). Compared to normal vessels, tumor vessels have an altered structure that leads to a limited supply of nutrients, form a hypoxic environment and alter the pH of the niche [17]. Hypoxia and acidic pH typical of the niche can create a barrier to drug distribution into the niches that can give resistance to chemotherapeutics, as shown by many authors for melanoma and other solid tumors [18,19,20].

The heterogeneous nature of the biology of solid tumors and the identification of prevalent single genetic mutations often lead to partial treatment opportunity and efficacy. The complex network of cancer cells and niche is an extremely interesting field of study where the integration of multiple diagnostic, genetic, cellular, and pharmacological data is mandatory, and where artificial intelligence (AI) might provide a significant contribution [21,22,23]. This narrative review describes the peculiarities of the niches in order to exploit chemical approaches to be used to improve the treatment with chemotherapeutics and provide insights for the development of new pharmacological strategies.

## 2. The Tumor Microenvironment Niche Complexity

**Figure 1:**
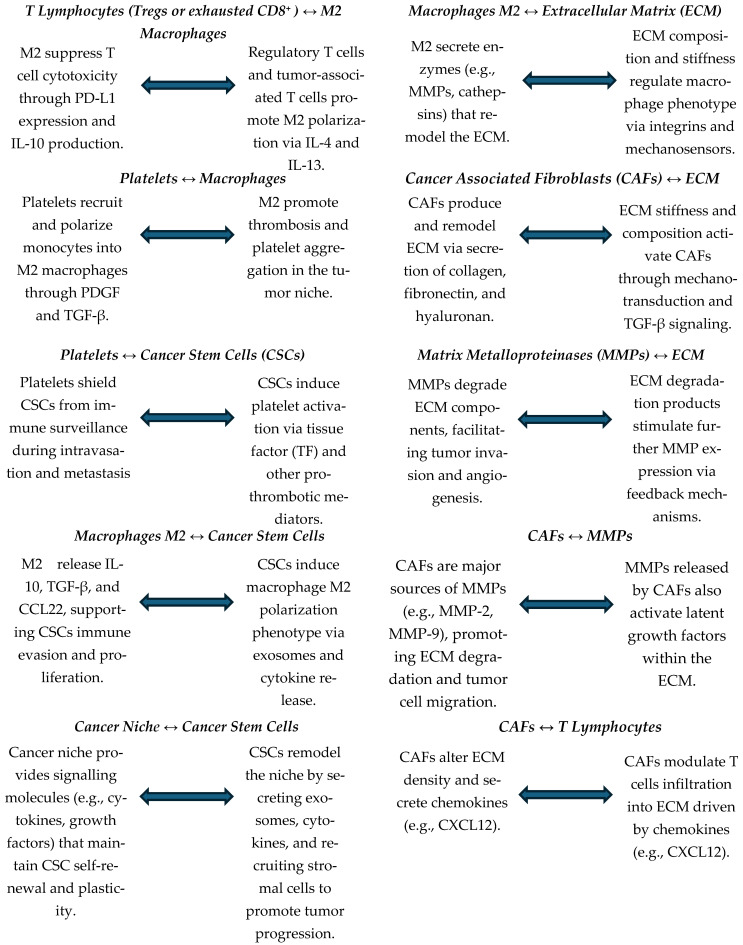
Schematic representation of the bidirectional interactions between the different components of the TME (in bold). TME = tumor microenvironment; CSCs = cancer stem cells; CAFs = cancer-associated fibroblasts; ECM = extracellular matrix; M2 = tumor-associated macrophages (TAMs); MMPs = metalloproteinases.

Solid and liquid tumors are a highly complex, heterogeneous, and dynamic ensemble of cell types that comprises heterogeneous malignant and non-malignant cells. Many authors have described the importance of fibroblasts, vascular endothelial cells, platelets, immune cells, such as macrophages and T-cells, and organ-specific interstitial cells as stable components in most tumors [5,24]. Furthermore, the niche is embedded in a modified extracellular matrix (ECM), composed of collagen, fibronectin, hyaluronan, and laminin, among other proteins [25,26,27,28,29]. Any of these components have shown their contributions to tumor formation, protection against the immune system, and support for metastatic development [30,31,32]. Collectively, all of these components, together with factors such as oxygen levels, pH, and the presence of many abundant molecules produced and released by cells, form a TME that provides all essential factors for cancer initiation and progression. The interactions between the different components of the TME have been shown to be bidirectional, since tumor cells can change the nature of the microenvironment and vice versa [33,34,35]. Among other factors, these bidirectional interactions involve exosomes, miRNA trafficking, and epigenetic modifications in cancer cells and somatic niche cells [36,37]. These interactions create a network of connections capable of promoting neoplasm progression by specific signals and mediators, most of which are still to be elucidated, and providing the exact stimuli and support for the spread of metastases (Figure 1) [38,39,40,41].

The concept of TME was anticipated by Stephen Paget in 1889 in the “seed and soil” hypothesis, elaborated to explain the mechanism of metastasis: “When a plant goes to seed, its seeds are carried in all directions; but they can only live and grow if they fall on congenial soil.” In other words, a “seed” of cancer cells requires a welcoming environment and “soil” to grow and develop [42].

The acquisition of intrinsic replicative autonomy of the tumor cell, as it is often linked to the appearance of random mutations, is also called “driver mutations”, which play a fundamental role in the early stages of neoplastic development, since they are associated with the dysregulation of oncogenes involved in the control of cell replication. Many authors suggest that at least five driver mutations must coexist to have the onset of neoplastic cells from an altered somatic cell [43]. The “passenger mutations”, instead, do not confer any substantial replicative or survival advantage to the tumor mass. These mutations are secondary and accidental, and generally affect genes defined as “tumor suppressor” and can lead to the etiological and evolutionary understanding of the tumor [44].

Traditional chemotherapy, which includes fundamental drugs such as the alkylating and platinating agents (e.g., cyclophosphamide, cisplatin), antimetabolites and analogues of the bases (e.g., methotrexate, 5-fluorouracile, gemcitabine), anthracyclines (doxorubicin), mitotic fuse blockers (taxans and vinca alkaloids), and hormone agonists and antagonists (e.g., goserelin, tamoxifen), has offered enormous successes against a number of liquid and solid tumors. However, there are limitations to its efficacy due to the heterogeneous composition of the tumor mass, especially in advanced tumor stages, and the lack of action on niche components. Mutated sub-populations can cause tumor relapses due to clonal selection of resistant cells. For the same reason, newer targeted therapies, such as the variety of monoclonal antibodies (whose name has a suffix -mab, -tug, -bart, -ment, or -mig), the tyrosine kinase inhibitors (drugs with the suffix -tinib, -fenib), and the cyclin-dependent kinase inhibitors (with the suffix -ciclib), although focused against a single target receptor that is overexpressed or mutated in cancer cells, can have low efficacy due to the evolution of resistant cells [45,46]. Furthermore, the treatment of metastases and their relationship with the primary tumor still represent a challenge for current antineoplastic therapy [46,47,48]. Once again, cancer treatment focused only on the “seeds” (i.e., the neoplastic cell), disregarding the effect of the “soil” (i.e., tumor niche), might limit the long-term success of therapy. On the contrary, acting against the major elements present in the niche in synergy with highly selective and traditional treatments might contribute to defining more efficient anticancer strategies [10].

## 3. The Niche Components for Integrated Pharmacological Cancer Treatments

### 3.1. Exploiting the PH as Activator of Prodrugs

Under physiological conditions, systemic pH is maintained between 7.35 and 7.45, while intracellular pH is generally approximately 7.2 despite continuous acid production by cell metabolism. The cell membrane embeds many different types of ion channels, transporters, and pumps that allow H^+^ and HCO_3_^−^ ions to move inside and outside of the cell. Given the molecular complexity in cell ionic homeostasis, several key intracellular pH-regulating systems, including the Na^+^/H^+^ exchangers, the proton pump, the monocarboxylate transporters, the HCO_3_^−^ transporters and exchangers, and the membrane-associated and cytosolic carbonic anhydrases (CAs), operate together to maintain pH values that are acceptable for cell survival. Intracellular and extracellular buffers are the most immediate mechanism of defense against changes in systemic pH, whereas alveolar ventilation and renal control of plasma HCO_3_^−^ make the rest. In normal tissues and cells, extracellular pH (pH_e_) is usually slightly higher than intracellular pH (pH_i_) (pH_e_ = 7.3–7.5 vs. pH_i_ = 6.8–7.2) [49].

Cancer cell metabolism is characterized by an increased uptake of glucose and, consequently, a high requirement for oxygen. However, in the different parts of the tumor mass, there is a heterogeneous delivery of nutrients and oxygen due to the presence of an irregular network of blood vessels, resulting in limitations of O_2_ availability and hypoxia. Cancer cells react to such adverse conditions through a series of molecular changes involving increased expression of nutrient and ion transporters and enzymes. For example, reduced oxygen levels activate the Hypoxia Inducible Factor-1α (HIF-1α) that up-regulates the transcription of glucose transporters and enzymes involved in glucose metabolism (e.g., 6-phosphofructo-1-kinase, PFK-1). Moreover, under hypoxic conditions, glucose is converted mainly to lactic acid through the glycolytic pathway to produce energy in the form of ATP (Warburg effect), further decreasing the pH of extracellular spaces [50]. The drop in pH also influences the activity of lactate and various ion transporters that collectively contribute to the recovery of intracellular acid homeostasis. Finally, hypoxia also causes the increased expression of some membrane-bound enzymes, such as Cas, that catalyze the hydration of CO_2_ to H^+^ and HCO_3_^−^ ions on the cell surface [51,52,53,54,55].

In conclusion, the combination of all the above-mentioned effects makes extracellular acidity a primary feature of TME. In fact, cancer cells develop a reversed pH gradient (pH_e_ < pH_i_) [56,57] with respect to normal cells (pH_e_ > pH_i_). Importantly, the acidic tumor pH_e_ (6.5–6.9) has been reported to create a physiological barrier to drugs that can represent a contribution to the resistance to chemotherapeutics (ion trapping effect) [58,59,60]. Molecules such as doxorubicin, anthracyclines, daunorubicin, mitoxantrone, and imidazoacridinones are uncharged weak bases and freely permeate membranes. However, in acidic solutions, these molecules are ionized, becoming positively charged species. This change reduces their permeability to cells, trapping them in extracellular compartments and reducing their cellular uptake and efficacy. On the contrary, weak acids tend to concentrate in more alkaline environments, such as tumor intracellular compartments [61,62,63].

Consequently, the acidic pH of TME may represent a valuable pharmacological trigger, but also an interesting target for designing diagnostic or theranostic molecules, producing imaging agents to study tumor metabolism, and sensors to measure treatment results [64].

The simplest way to obtain pH-triggered molecules is through the design of inactive low molecular weight prodrugs containing a masked pharmacophore activated only under acidic pH tumoral conditions. Alternatively, nanoparticles may offer a rich and diverse palette of materials that remain stable and retain their chemotherapeutic cargo in the cellular niche until exposure to an appropriate stimulus (i.e., low pH) [65,66,67,68,69].

For example, at neutral pH, ferromagnetic (γ-Fe_2_O_3_ or Fe_3_O_4_) nanoparticles (NPs) can catalytically break down H_2_O_2_ into non-toxic H_2_O and O_2_, while in the acidic TME, they generate reactive oxygen species (ROS) such as hydroxyl radicals (^λ^OH) from H_2_O_2_, which induce apoptosis and tumor death. More complicated structures can be exploited to combine different features. For example, ultrasmall Fe_3_O_4_ nanoparticles and glucose oxidase (GOD) were loaded into biodegradable dendritic silica nanoparticles. GOD catalyzes the oxidation of glucose, producing H_2_O_2_, which is in turn transformed into highly toxic hydroxyl radicals for subsequent Fenton-like reaction catalyzed by Fe_3_O_4_ nanoparticles in mild acidic TME [70,71].

Different NPs can act as carriers for drugs to be released under acidic conditions. The pH-sensitivity and possibility of functionalization demonstrated by CaCO_3_ NPs make them promising drug carriers for cancer tissues and cells, although no solid data from clinical trials are available yet. Actually, in the acidic TME, CaCO_3_ NPs release Ca^2+^ and CO_2_ together with their possible payload, such as doxorubicin, photosensitizers, or small interfering RNAs (siRNAs) [72]. Moreover, due to the slow degradation of CaCO_3_, these NPs can be used for prolonged release, thus retaining drugs in cancer tissues for longer times after administration [70,71,73].

**Figure 2:**
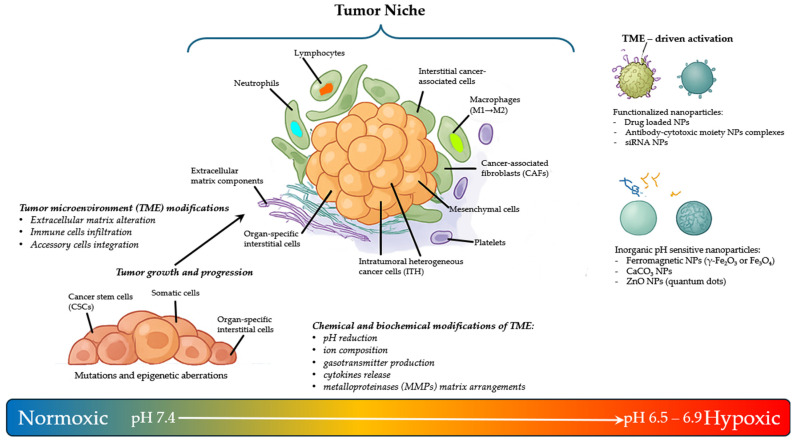
Schematic representation of the evolution of TME with niche formation. Cancer evolution and progression to metastases are sustained by TME modification and cells of different origin composition. TME chemical and biochemical properties (e.g., hypoxia and acidification) can promote the selective activation or release, like functionalized and pH-sensitive nanoparticles. TME = tumor microenvironment; CSCs = cancer stem cells; CAFs = cancer-associated fibroblasts; ITHs = intratumoral heterogeneous cancer cells; M1 = pro-inflammatory macrophages; M2 = tumor-associated macrophages (TAMs); MMP = metalloproteinase; siRNA = small interfering RNA; NPs = nanoparticles.

Similarly, pH-sensitive ZnO NPs (quantum dots) dissolve in acidic endosomes/lysosomes in cancer cells (pH < 5.5), triggering the controlled release of loaded drugs. ZnO quantum dots with surface modified by amino groups were the platform for the introduction of polyethylene glycol (PEG) and hyaluronic acid molecules. To this core, NP doxorubicin was loaded not only by covalent interactions but also by the formation of Zn^2+^−doxorubicin chelate complex [74,75] (Figure 2).

Also, in the field of organic molecules, efficient drug carriers for drugs can be designed, because their properties can match the TME. In polymers that contain pH-sensitive groups (e.g., amino, imidazolyl, carboxyl groups), the protonation at acidic pH destabilizes the structure, causing the release of non-covalently loaded drugs. Acid-labile chemical bonds (e.g., ester, acetal/ketal, hydrazone) linking the delivery systems to the payload are also used for pH-triggered drug release [76,77]. For example, doxorubicin conjugated via the acid-labile hydrazone bond onto hydrophobic segments of the folate-conjugated micelles based on amphiphilic hyperbranched block copolymer, Boltorn^®^ H40-poly(L-aspartate-doxorubicin)-b-poly(ethylene glycol)/folate-conjugated poly(ethylene glycol), was released rapidly at typical acidic TME pH [78]. 

Another strategy exploits pH-sensitive groups (e.g., diorto esters, vinyl esters, cystein-cleavable lipopolymers, double esters, hydrazones) to detach coatings of drug delivery systems, such as PEG. Detachment of PEG due to acidified pH could facilitate interaction with the cell and/or the release of the encapsulated drug/DNA [77,79]. For example, low MW polyethyleneimine (PEI) modified with dioleoylphosphatidylethanolamine (PE) formed an efficient gene delivery system. The resulting conjugate PEI-PE/DNA was mixed with low-pH degradable PEG–hydrazone–PE co-polymer, thus producing particles with transfection activity sensitive to the relatively acidic pH of TME. The high transfection efficacy at acidic pH is due to PEG detachment and efficient uptake of remaining positively charged DNA complexes by cells [80]. Clinical data from pH-sensitive prodrugs are not conclusive and show some limitations. Major limitations have been the in vivo compatibility and toxicity of cross-linking agents or monomers, plus the achievement of targeted accumulation of drugs. Some authors proposed multifunctional approaches to overcome some of these limitations, like for example the pH-responsive epigallocatechin gallate (EGCG)-conjugated low-molecular-weight chitosan (LC-EGCG, LE) nanoparticle (Met-GOx/Fe@LE NPs) and curcumin (Cur) and glucose oxidase (Gox) into a lipid copper-based organic framework (LCOF) or many others, and preliminary in vivo animal studies are promising [81,82].

### 3.2. Exploiting the Hypoxia in the Niche as a Trigger for Activating Drugs

A reduced O_2_ content is also a source of stress for the cancer cell that may undergo two processes: slowing its progression and proceeding to necrosis/apoptosis or adapting to these unfavorable conditions [60,83,84].

The term “normoxia” is generally used to describe normal oxygen levels in tissue culture flasks (i.e., the O_2_ content in the air, approximately 20–21% *w*/*w* oxygen). Of course, this is not the content of O_2_, neither in the lung alveoli (about 14.5%) nor in the arterial blood (9.5%) or at the venous end of the circulation (6.5%). The term “physioxia” has been proposed to indicate the physiological oxygen level in peripheral tissues (5%), so that physiological “hypoxia” can be observed below 2% O_2_ (=15 mmHg at 37 °C) [85].

The adaptation process to unfavorable conditions is primarily orchestrated by hypoxia-inducible transcription factors (HIFs). In particular, HIF-1 forms heterodimers composed of HIF-1α (or its analogs HIF-2α and HIF-3α) and HIF-1β subunits. Different from HIF-1β, which is constitutively expressed, HIF-1α has a short half-life t_½_ of less than 5 min under normal conditions. Under reduced oxygen, HIF-1α is stabilized and translocates to the nucleus, where it dimerizes with HIF-1β, forming activated HIF-1. On the contrary, HIF-2α can be detected near physioxia, and the role of HIF-3α is still largely unclear. Interestingly, HIF-1α but not HIF-2α appears to regulate extracellular acidification in hypoxic tumors. HIF-1 induces up to 200 genes that promote adaptation to hypoxia, supporting cell survival by stimulating compensatory angiogenesis, remodeling of the ECM, metabolic shift, and immune suppression 45–52 [86,87,88,89,90,91,92,93]. More importantly, the HIF-regulated gene network has been shown to promote physiological changes associated with therapeutic resistance, including inhibition of apoptosis and senescence and activation of drug efflux and cellular metabolism [94,95,96].

The relationship between HIF-1α overexpression and cell proliferation has been observed in many human cancers, as well as the relationship between HIF-1α accumulation and shorter survival in patients with early-stage cervical, breast, ovarian, endometrial, oropharyngeal squamous cell carcinoma, and oligodendroglioma. Significant associations have been reported between HIF-2α overexpression and increased patient mortality for non-small-cell lung cancer, neuroblastoma, astrocytoma, and head and neck squamous cell carcinoma [97].

Hypoxia-modified pH gradients (see Section 3.1) can alter the activity of pH-dependent chemotherapeutics (such as doxorubicin and docetaxel) and DNA alkylating agents (such as temozolomide, chlorambucil, and ifosfamide) [98]. Some authors have proposed multimodal molecules to increase tumor sensitivity in the cancer niche by mitigating hypoxia and acidic conditions [99]. In addition, in vitro hypoxia-induced drug resistance has been reported for 5-fluorouracil, adriamycin, docetaxel, doxorubicin, etoposide, flutamide, gemcitabine, methotrexate, *N*-(4-hydroxyphenyl)retinamide, paclitaxel, platinum(II)-based drugs (i.e., carboplatin, cisplatin, and oxaliplatin), and vincristine, as a single drug or in combination, due to HIF-1-mediated regulation of drug efflux, alterations in cell proliferation and survival, inhibition of DNA damage, and metabolic reprogramming [95].

Therefore, HIF-1 inhibition has been proposed as a rational target for cancer therapy, in an attempt to induce unfavorable conditions of TME in tumor niches for cancer cell survival and drug resistance development [100,101]. In addition to leveraging hypoxia as a chemical trigger to activate prodrugs, an alternative strategy is to directly inhibit the cellular adaptation mechanisms that sustain cancer survival in hypoxic conditions. HIF-1 signaling pathway drives angiogenesis, metabolic shifts, and resistance to therapy. Inhibiting HIF-1α or its upstream regulators represents a pharmacological approach aimed at dismantling the tumor’s ability to thrive under low oxygen pressure. HIF-1 inhibition can be achieved through the most important HIF-1α regulatory systems, such as PI3K/Akt/mTOR, MAPK, the Hsp90 system, the enzymatic complexes Topo-1 isomerase and thioredoxin-1 redox protein [102]. For example, among the molecules able to downregulate HIF-1α protein by interfering with the PI3K/Akt pathway (an intracellular pathway important in regulating the cell cycle, also related to proliferation and cancer), SU5416 (semaxanib) is an oxindole derivative that reversibly inhibits ATP binding to the tyrosine kinase domain of vascular endothelial growth factor (VEGF) receptor. This compound makes the antiangiogenic effect of radiotherapy easier. It has been tested in clinical trials up to phase III in combination with chemotherapy against advanced metastatic colorectal carcinoma and adenocarcinoma, with no definitive results. SU5416 produces a decrease in HIF-1α protein and VEGF mRNA through the PI3K/Akt pathway in ovarian carcinoma cells [103]. 

Another approach to the downregulation of HIF-1α is inhibition of farnesyl transferase to inhibit, in turn, mitogen-activated protein kinase (MAPK, also involved in the regulation of proliferation, gene expression, cell survival, and apoptosis) pathway activators Ras and Rho. Several farnesyl transferase inhibitors have been tested in clinical trials for various kinds of cancer but only tipifarnib and lonafarnib have been evaluated in phase III clinical trials in hematological and solid tumors [104,105]. 

Hsp90 is a chaperone assisting other proteins that are also required for tumor growth. It also plays a role in HIF-1a protein folding, protects against the degradation of HIF-1α protein by the proteasome, and stabilizes the interaction between HIF-1α and ARNT, regulating HIF-1α transcriptional activity [106]. For example, the antibiotic geldanamycin binds to Hsp90 and has been shown to reduce HIF-1α protein levels via its effects on Hsp90, and to inhibit HIF-1α transcriptional activity. Some derivatives of geldanamycin, such as 17-(allylamino)-17-demethoxygeldanamycin (17-AAG) and 17-dimethylaminoethylamino-17-demethoxygeldanamycin (17-DMAG), have been evaluated in clinical trials, although more recently small molecules that prevent assembly of the complex have been proposed as an alternative method of Hsp90 inhibition due to their lower adverse drug reactions [107]. 

Another way to exploit low O_2_ conditions is through the design of molecules selectively activated under hypoxic stress. Recently, hypoxia-activated prodrugs underwent clinical trials. Among those, evofosfamide (TH-302) showed interesting features, since it is reduced under hypoxic conditions to produce cytotoxic metabolites. Interesting activities have been shown in acute myeloid leukemia, nasopharyngeal carcinoma cells, and prostate cancer by increasing sensitivity to immune checkpoint blockers and normalizing the tumor vascular system. Phase III clinical trials showed some limitations in bioavailability; thus, some combinations with other drugs and chemical modifications have also been proposed, like monomethoxy polyethylene glycol-Poly(lactic-co-glycolic acid) (mPEG-PLGA)-encapsulated TH-302 nanoparticles (TH-302 NPs) [108,109].

Azobenzene was used as a hypoxia-responsive linker between PEG and PEI in lipid–PEI–PEG copolymer micelles containing 1,2-dioleoyl-*sn*-glycero-3-phosphoethanolamine (DOPE) and loaded with siRNA. The hypoxic and reductive tumor environment causes specific detachment of PEG due to degradation of the azobenzene linker. Moreover, the remaining positively charged PEI–DOPE/siRNA conjugates can be effectively taken up in the cell [77,110].

Since multiple factors (e.g., hypoxia, acidosis, ROS) must be faced in TME, a single chemical with multiple effects can be more potent. In MnO_2_ NPs, MnO_2_ shows high specificity and reactivity toward H_2_O_2,_ producing O_2_ and H_2_O without generation of ^λ^OH, and consuming protons. The consequence is the breakdown of MnO_2_ to harmless Mn(II) ions, thus avoiding toxicity caused by accumulated NPs. Thus, MnO_2_ NPs proved to be effective in niches by reducing endogenous H_2_O_2_ levels, reducing hypoxia by generating O_2_ in situ, and reducing acidosis by consuming protons. Moreover, complex hybrid NPs were considered to improve hydrophobicity and structure suitable for intravenous injection, with optimal biocompatibility, high tumor accumulation, and programmable oxygen generation rate [70,111,112]. Some interesting clinical translations of agents that are activated by acidosis are described in the literature. These molecules have shown improved selectivity and targeting. For example, ONM-100, a nanoparticle-based fluorescent imaging agent, is an ultra-pH sensitive amphiphilic polymer, conjugated with indocyanine green, which dissociates to fluoresce in the acidic TME [113]. Other approaches are therapeutic vesicular nanoreactors like glucose oxidase (GOD)-loaded therapeutic vesicular NRs (theraNR) that are copolymers containing poly (ethylene glycol) (PEG) [113,114]. The preliminary results are quite interesting and show clear possible clinical applications.

### 3.3. Exploiting the Potential of Metal-Based Drugs for Tumor Niche Modification

In line with the concept of using tumor niche features as chemical triggers, redox-active metal-based drugs represent an opportunity to selectively deliver or activate therapeutic payloads within the TME. Specifically, Pt(IV) complexes exploit the hypoxic and reductive conditions commonly found in tumor niches to undergo intracellular activation. This section explores how such compounds can complement strategies based on pH and hypoxia modulation by serving as smart, redox-responsive prodrugs. Redox metal complexes represent an alternative to organic molecules in the modulation of biological systems. Some metal ions can exist in different oxidation states, characterized by different reactivity toward biomolecules, so that TME and complex exert a mutual influence [115,116].

**Figure 3:**
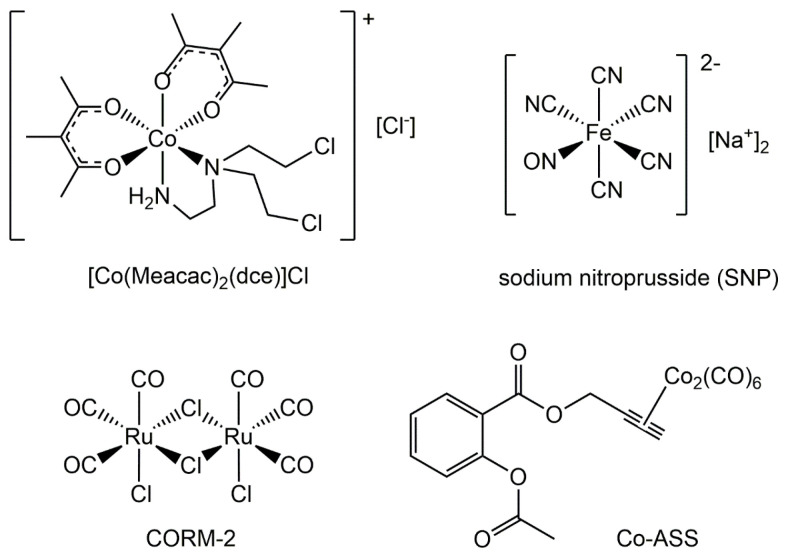
Structural representations of a few selected metal complexes cited in the paper: Co(III) complex with a nitrogen mustard [Co(Meacac)_2_(dce)]Cl (Meacac = methyl acetylacetonate, dce = N,N-bis(2-chloroethyl)ethylenediamine); sodium nitroprusside (SNP); tricarbonyldichlororuthenium(II) dimer (CORM-2); [2-acetoxy-(2-propynyl)benzoate]hexacarbonyldicobalt (Co-ASS).

**Figure 4:**
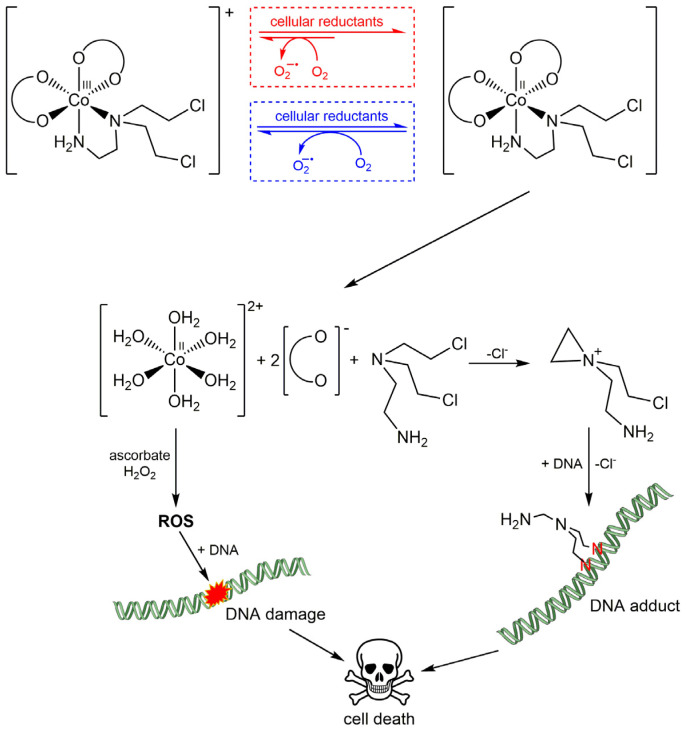
Schematic and simplified behavior of [Co(Meacac)_2_(dce)]Cl (Meacac = methyl acetylacetonate, dce = N,N-bis(2-chloroethyl)ethylenediamine) in hypoxic vs. normoxic environments (the two environments are highlighted in red and blue, respectively). In hypoxic cells, the Co(III) reduction prevails over the Co(II) reoxidation. Cellular reductants that could help the reduction process are DT diaphorase, xanthine dehydrogenase, nitroreductase, and cytochrome P450 reductase. After reduction, the Co(II) intermediate leads to aquation, forming the Co(II) species that may start a Fenton-like reaction, producing DNA-damaging reactive oxygen species (ROS) and, hence, bringing cells to death. The released nitrogen mustard is able to alkylate DNA bases (N7 position of guanines to form interstrand cross-links), which results in the impairment of DNA replication and transcription, leading to cell death.

One of the first examples is represented by Co(III) complexes containing nitrogen mustard ligands, such as mechlorethamine and its derivatives, which have been widely used as anticancer drugs since the discovery of their antitumor effect in 1942. Co(III) complexes are kinetically inert, inhibiting the premature release of the ligands in normal oxygenated cells. In hypoxic solid TME, the metal center can be reduced, forming the kinetically labile Co(II), which can release the active mustard ligand (Figure 3). Following its release, the free lone pair of nitrogen mustard would form an aziridinium ion and then alkylate biological substrates (i.e., DNA, RNA, and proteins). In contrast, in a normoxic cell, O_2_ would reoxidise Co(II), preventing the release of the active mustard. But cobalt is not simply a *chaperone* that can selectively deliver nitrogen mustard. Co(II) ions themselves induce the generation of cytotoxic ROS in a Fenton-like reaction (Figure 4). The fine-tuning of the redox properties, which is fundamental to selectivity, has proved to be quite difficult, and only a few Co(III) and Cu(II) complexes have shown potential in this field, but none of them have been developed at the clinical level so far [115,117,118]. 

**Figure 5:**
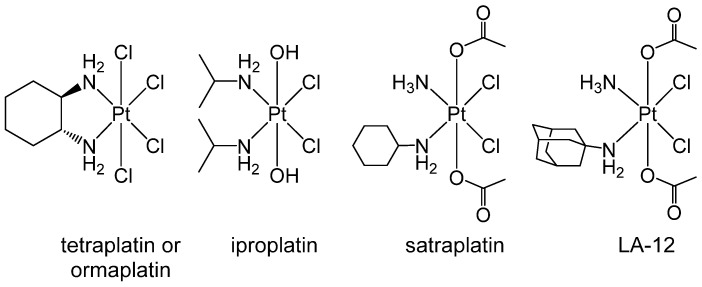
Octahedral Pt(IV) complexes exploited for antiproliferative strategies.

A family of metal compounds that showed some interesting features in terms of the exploitation of TME is represented by the octahedral Pt(IV) complexes. These compounds are actively studied as an alternative to cisplatin-like Pt(II) congeners because they exhibit higher kinetic inertness, decreasing the incidence of other undesirable reactions, limiting side effects, and making them also suitable for oral administration (Figure 5).

**Figure 6:**
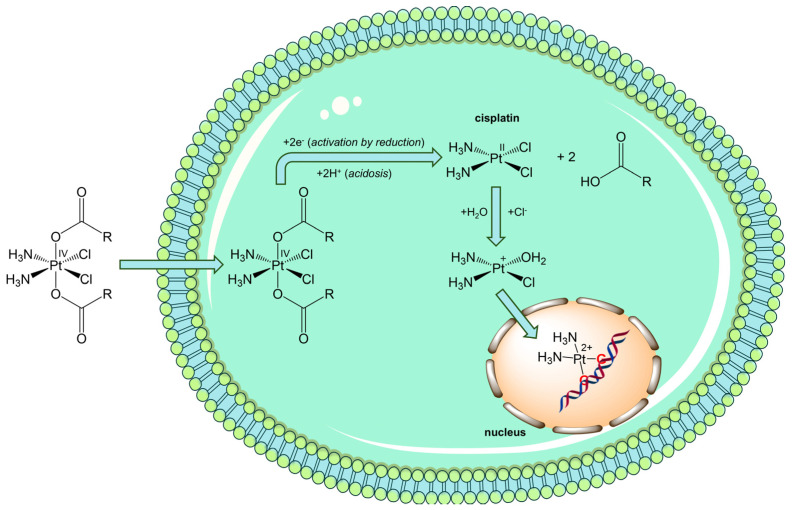
Simplified scheme of the 2e^−^ reduction of a cisplatin-based dicarboxylato Pt(IV) complex (*activation by reduction*) followed by the formation of DNA adducts between Pt(II) species and DNA, leading to cell death, mainly by apoptosis.

It is noteworthy that these compounds are not deactivated by hypoxia, as in the case of Pt(II) drugs but, on the contrary, they can exploit hypoxic TME. In fact, Pt(IV) complexes should reach cancer cells intact, where they are activated by reductive elimination of axial ligands to release the cytotoxic Pt(II) complex, thus acting as prodrugs activated by reduction (Figure 6). Furthermore, the reduction reaction is favored by acidic TME by protonation of the released axial carboxylates [119,120,121].

The formed metabolite (for instance cisplatin, (*SP*-4-2)-diamminedichloridoplatinum(II)) undergoes aquation to form [PtCl(NH_3_)_2_(OH_2_)]^+^ and [Pt(NH_3_)_2_(OH_2_)_2_]^2+^ once inside the cell. The platinum atom covalently binds the N7 positions of purine bases to afford primarily 1,2- or 1,3-intrastrand crosslinks. Cisplatin modifications distort the structure of the DNA duplex and these lesions, recognized by high-mobility group (HMG) box proteins, repair proteins, transcription factors, and other proteins, cause various cellular responses, such as replication arrest, transcription inhibition, cell-cycle arrest, and, finally, apoptosis. Moreover, it has been demonstrated that cisplatin can influence the TME because it can severely affect the intracellular redox homeostasis by disruption of the thioredoxin reductase (TrxR) and glutathione reductase (GSR) systems [115,122,123].

Over the years, few Pt(IV) complexes progressed to clinical studies, although research in this field remains very active [124]. Ormaplatin or tetraplatin, (*OC*-6-22)-tetrachlorido(cyclohexane-1*R*,2*R*-diamine)platinum(IV), showed severe and unpredictable cumulative neurotoxicity, while the activity of iproplatin ((*OC*-6-33)-bis(hydroxido)dichloridodiisopropylamineplatinum(IV)) showed objective response like cisplatin or carboplatin. LA-12 ((*OC*-6-43)-bis(acetato)(1-adamantylamine)amminedichloridoplatinum(IV)) has entered clinical trials, but there are no published findings from phase I/II. To date, satraplatin ((*OC*-6-43)-bis(acetato)amminedichloridocyclohexylamineplatinum(IV)) is the most successful Pt(IV) prodrug that progressed to phase III clinical studies. However, the overall survival rate did not improve significantly, and more satraplatin trials are still ongoing [125,126].

More recently, Pt(IV) conjugates bearing bioactive carboxylate molecules as axial ligands have been designed. These functionalized prodrugs can contain specific tumor-targeting molecules (steroids, folates, amino acids and peptides, modified glucose or albumin, etc.), complementary pharmaceutical agents (very often enzyme inhibitors), or anticancer drugs that have targets different from DNA, capable of synergizing with the action of the cytotoxic Pt(II) metabolite released after reduction [127,128]. Since both Pt(II) drugs and bioactive ligands (especially those having epigenetic propensity) can act on different targets and with multiple cellular mechanisms, the final result is to have multi-action Pt(IV) prodrugs with better performances compared to the single fragments composing the molecules [129,130,131,132,133,134,135]. This field of research is quite active; a very recent review article lists more than 110 Pt(IV) compounds containing bioactive ligands that have been reported to date, and this number is increasing rapidly, including nanoparticle strategies [132,136]. Finally, as in the case of pH-triggered drugs, hypoxic niches may represent not only a valuable pharmacological trigger but also a diagnostic tool [137,138,139,140,141,142,143,144].

### 3.4. Can Gasotransmitters Disrupt Tme’s Support to Tumor Cells?

The tumor microenvironment’s biochemical profile also enables the use of gasotransmitters, such as NO and CO, as stimuli-responsive agents. These molecules can modulate signaling pathways and release therapeutic agents in response to oxidative stress, hypoxia, or enzymatic activity within the niche. This section focuses on the use of gasotransmitters as tools to fine-tune drug activity and delivery in TME conditions. Gasotransmitters define a family of small gaseous endogenous molecules that serve to regulate normal and pathological cellular activities. This family of molecules includes nitric oxide, hydrogen sulfide, carbon monoxide, and possibly sulfur dioxide and other gases [145,146,147,148,149].

Nitric oxide (NO) is a pleiotropic regulator that exhibits pro and antitumorigenic effects, which range from intracellular signaling to transcellular messenger, and the formation of cytotoxic species depending on the cell that releases it and its concentration [150,151]. Nitric oxide is produced by the conversion of L-arginine to L-citrulline by nitric oxide synthases (NOSs). This family of enzymes exists as three isoforms, which are the calcium-dependent endothelial (eNOS) and neuronal (nNOS) NOS, and a calcium-independent inducible NOS (iNOS), important for early immune defense and associated with malignant diseases [152]. However, the role of iNOS during tumor development is highly complex and not fully understood. Both promoting and deterring actions have been described, presumably depending on the local concentration of iNOS within the tumor microenvironment. In fact, NO can affect cancer initiation and progression, angiogenesis, apoptosis, and tumor suppression, depending on both its concentration and duration of its action. Even though the exact mechanism of action is unknown, at low (50–100 nM) and intermediate (100–400 nM) concentrations, NO can stimulate cancer progression, prevent apoptosis, and enhance angiogenesis and metastasis. Higher NO concentrations (400–1000 nM) can induce apoptosis and sensitize tumors to chemo and radiotherapy. Moreover, NO is a strong ligand in metal complexes, above all iron complexes; therefore, NO can regulate many signaling pathways through its binding to metal centers (e.g., heme group and iron-sulfur clusters) [153].

Organic nitrates are the oldest and most widely used NO-releasing molecules (NORMs) and data on the effect of their influence on TME are controversial. The most widely employed ones to prevent angina symptoms and treat heart failure are glyceryl trinitrate, isosorbide mononitrate, pentaerythritol tetranitrate, and nicorandil. Many members of this family of compounds are highly stable, even though the NO release can happen via both enzymatic and non-enzymatic pathways. A second class of NO-releasing molecules is represented by organic nitrites, where the nitrosyl nitrogen atoms are highly electronegative and prone to nucleophilic attack. In this case, the release of NO occurs through *S*-nitrosothiol formation from fast nitrosation of thiols. To avoid the nitrosation step, *S*-nitrosothiols or thionitrites have been directly studied as NO donors. Unfortunately, they are generally unstable in aqueous solution due to the cleavage of the S-NO bond to give the corresponding disulfide and NO, NO^+^, or NO^−^. Their degradation in vivo is increased by the presence of ascorbic acid, metal ions, thiols, oxygen, or by enzymatic cleavage [154].

As far as NO can bind to metal ions in vivo, metal-nitric oxide complexes can be used as NO donors. NO forms complexes with transition metal ions with a very short M-N bond, indicating the electronic structure M≡NO. Formally, such a bond is made of one σ-bond, involving the nitrogen lone pair and available metal σ-orbitals, and two π-bonds, involving metal d-orbitals and the degenerate π*NO molecular orbital of relatively low energy. The character of M–(NO-π*) bonds may vary between an M→NO^+^ backbond and an M←NO^−^ donor-bond, being the origin of the ambiguities when determining the oxidation-state of the metal centre and the mechanism of NO release [155,156,157]. 

NO in sodium nitroprusside (SNP, Na_2_[Fe(CN)_5_NO]) exhibits a significant NO^+^ character and iron is present as Fe^3+^. Thus, the NO ligand can undergo nucleophilic attack by thiols or other nucleophiles. The ability to release NO relatively quickly (circulatory t_½_ about 2 min) makes SNP a clinical vasodilator in cardiac surgery, hypertensive crisis, heart failure, and vascular surgery. However, together with NO, cyanides are released as well, thus excluding long-term administration, as requested in cancer treatment. Ruthenium also has a high affinity for nitric oxide and, for this reason, NORM Ru complexes have been studied in vitro and in vivo for controlled NO delivery in solid tumors. However, efficient NO release requires photoactivation with high-power UV light (300–400 nm), which prevents its clinical use [154].

Short half-lives, non-specific NO release, and rapid clearance of most NORMs limited their use as anticancer drugs. Therefore, to overcome these limitations, much effort has been made to develop hybrid NO donor drugs by linking NO donor moieties to anticancer drugs. For example, NO-releasing nonsteroidal anti-inflammatory drug (NSAID) derivatives have been exploited for cancer therapy. NO-releasing topoisomerase inhibitors have also been synthesized by conjugation of the anticancer drug doxorubicin with a phenylsulfonyl furoxan moiety. These doxorubicin analogs accumulate in doxorubicin-resistant human colon cancer cells and result in high cytotoxicity. NO releasing histone deacetylase inhibitors have been reported as an improved version of the original inhibitor drug, against the human erythroleukemia cell line [154]. High concentrations of NO induce apoptosis and sensitize tumors to chemo and radiotherapy [158]. The mechanisms of action are not fully understood, but high, non-physiological levels of local release of NO are needed for p53-induced apoptosis. Furthermore, high concentrations of NO act by nitrosating or oxidizing thiol centers of proteins and inhibiting mitochondrial respiration, while in metastasis, epithelial cells lose normal cell-cell adhesion and gain mesenchymal markers, which promote cell migration and invasion (EMT = epithelial to mesenchymal transition). On the other side, leaky tumor vasculature, slow venous return, and poor lymphatic clearance thus lead to the accumulation of macromolecules within the tumor, like nanocarriers and PEG-conjugated molecules, thereby improving delivery of the drugs to the tumor (EPR = enhanced permeability and retention) [159,160]. Infusion of isosorbide dinitrate (ISDN) into the local feeding artery of tumors has been shown to enhance site-specific delivery of anticancer NPs in humans [161]. Several clinical studies have used NO donors such as GTN to improve the responsiveness of tumors to chemo and/or radiotherapy. Some examples are cisplatin and irinotecan plus isosorbide mononitrate (ISMN), 5-FU and radiation plus glyceryl trinitrate (GTN) patch, combination of vinorelbine and cisplatin plus GTN, Vinorelbine, cisplatin, and concurrent radiotherapy plus GTN patch [154].

Another strategy for controlled NO release in the tumor niche exploits the encapsulation of NO donor drugs within particles. For example, the ruthenium nitrosyl complex *trans*-[Ru(NH_3_)_4_(NO)(py)](BF_4_)_3_●H_2_O (py = pyridine) was loaded into poly-lactic-co-glycolic acid microparticles. The conjugate is phototoxic upon light irradiation due to NO release from the Ru complex [162].

Another example of a photoactive NO-releasing conjugate was obtained by loading a *S*-nitrosothiol into a co-polymer of styrene and maleic acid. Encapsulation lent the water solubility and protected it from degradation. Photoactivation of the *S*-nitrosothiol group enhances the NO release from t_½_ of 104 h to 3.5 min. When the conjugate was co-administered with NPs loaded with doxorubicin, the anticancer properties of doxorubicin were significantly enhanced [154,163].

Moreover, to improve the NO release in the tumor niche, a wide variety of NO donor materials such as macromolecule scaffolds, polymeric NPs, micelles, dendrimers, sol-gel derived silica NPs, and metal oxide NPs have been studied as NO delivery systems to treat a large number of pathological conditions, also including cancer [154].

Another possibility to influence niches is to drive the immune cells present in the tumor mass, such as macrophages and neutrophils. In immune cells, iNOS produces high concentrations of NO using L-arginine (L-Arg) as a substrate. Poly(ethylene glycol)-block-poly(L-arginine) (PEG-*b*-P(L-Arg)) block copolymers were prepared and used with chondroitin sulfate to prepare micelles (PEG-*b*-P(L-Arg)/m) for anticancer immunotherapy directed directly into TME. RAW264.7 adherent cell line of monocyte/macrophages isolated from a mouse tumor was able to generate NO from the PEG-*b*-P(L-Arg)/m. Moreover, systemic administration of PEG-*b*-P(L-Arg)/m had no noticeable adverse effects and suppressed the tumor growth in C26 tumor-bearing mice [137].

Finally, several studies focused on carbon monoxide (CO) as a gasotransmitter, pointing out its anti-inflammatory, anti-apoptotic, cytoprotective, antiproliferative, and vasodilatory effects. As an inert gaseous molecule, CO is very stable and freely diffuses into all the cells. The main endogenous source of carbon monoxide is the degradation of heme into biliverdin, iron, and CO, catalyzed by heme oxygenases (HOs). Due to its product CO, the inducible isoform of HO, HO-1, has been reported to have protective effects against oxidative stress, inflammation, and cell cycle dysregulation [164,165].

The simplest approach to the delivery of CO into the body would be its inhalation. However, due to its toxicity, CO must be administered in a strictly controlled way. Moreover, there is a lack of specificity between healthy and pathological tissues when gaseous CO is distributed through circulation, and this distribution is further complicated by the relatively low water solubility of CO. Since translocation of CO through the body is difficult, systems of controlled delivery of CO are needed [165,166,167]. Carbon monoxide-releasing molecules (CORMs) are prodrugs for physiological CO release. Common CORMs contain a transition metal (e.g., manganese, ruthenium, molybdenum, iron) and carbonyls as ligands. There are three main mechanisms for the release of CO: ligand-exchange, enzymatic reaction, and photochemical activation. Other CORMs are boranocarbonates, containing the metalloid boron, and non-metallic CORMs. For example, tricarbonyldichlororuthenium(II) dimer (CORM-2) rapidly releases CO in physiological buffers (t_½_ = 1 min, 37 °C, pH 7.4) (Figure 2). It is insoluble in water and requires the addition of dimethyl sulfoxide (DMSO) that promotes the release of CO via ligand-exchange, resulting in a series of inactivated CORMs containing DMSO [168]. CORM-2 was widely employed in in vitro and in vivo studies. For example, it was evaluated in an orthotopic allograft lung cancer mouse model. Controlled release of CO from CORM-2 at physiological concentration suppressed the inflammation reaction and protein synthesis signaling, inhibiting aberrant cell proliferation and malignant growth [169]. CO, in the form of CORM-2 or CO gas, exerted a potential antiproliferative effect on a panel of human pancreatic cancer cell lines, in a dose-dependent manner. CORM-2 treatment led to an increase in the survival rate of in vivo pancreatic cancer models with a significant decrease in the tumor volumes at 35 mg/kg/day [170]. The pharmacology of these compounds is difficult and more extensive studies are needed to exploit the real therapeutic potential of these molecules and the effects on niche modulation of cancer cells [171]. 

The antiproliferative activity of the hybrid CO-NSAID agent, a CO-releasing molecule retaining the NSAID aspirin, the [2-acetoxy-(2-propynyl)benzoate]hexacarbonyldicobalt (Co-ASS) (Figure 3), was investigated on malignant pleural mesothelioma (MPM) cells. Co-ASS induced cell death in sarcoma cell lines through the intrinsic apoptotic pathway, associated with a strong NF-κB inhibition, and showed only a modest cytostatic activity against epithelioid MPM cells. Co-ASS significantly reduced reactive oxygen/nitrogen species, suggesting that it releases CO, acting as a CORM [172]. 

To help contextualize the various strategies discussed, Table 1 provides a comparative overview of the major tumor niche-targeted approaches, including their mechanisms of action, specificity, stage of development, and translational challenges.

Cancer biology is offering new perspectives for pharmacological choices, but tumor microenvironment-responsive delivery systems under clinical trials have often been hampered by off-target toxicity, immunogenicity, low bioavailability, and biocompatibility that have limited successful translation into clinical practice. Despite this, there is a growing number of clinical protocols that have been developed and are currently ongoing. In fact, to date, there are 252 clinical trials listed on ClinicalTrials.gov (updated to June 2025) that involve TME studies. In addition to the above-mentioned molecules and those already used in the clinical setting, we can mention the most representative trials, which are summarized in Table 2. The intervention strategies against TME are often indirect and offer the possibility of integration with agents that address the peculiarities of TME. For most clinical trials, results are not yet available, but the FDA has approved some pH-responsive micellar formulations. The most representative is Genexol-PM (Samyang Co., Seoul, Korea), a formulation of polymeric micellar paclitaxel and diblock copolymers as solubilizer, that has reached clinical trials and received FDA approval for breast cancer treatment [173].

## 4. Conclusions

In recent years, substantial progress has been made in understanding the biological and cellular nature of tumor niches. The growing importance of the tumor niche as a pharmacological target for precise oncology treatments will push the production of new molecules with original therapeutic potential. Literature points out the importance of considering TME as an integral part of the tumor and not an entity independent from the fate of the neoplasm itself. The specific chemical and biochemical peculiarities of tumor niche described by the literature will pone the basis for the design of new drugs or multifunctional molecules. 

Further developments in niche understanding will be fundamental for developing new antineoplastic drugs and precise delivery and chemistry will contribute strongly to this process. To date, there are some limitations on the clinical development of niche-selective drugs. The biology of niches is complex and not fully understood, and the cell composition and function vary during the stages of cancer development. While combination therapy is the actual resource to target cancer cells and the support microenvironment, the analysis of many factors performed by artificial intelligence support will make the difference soon. Despite great developments in in vitro and in vivo animal models, preliminary clinical trials of TMS-directed drugs presented hurdles and limitations such as low response rates, failure to determine clinical effectiveness, and possible complications, such as autoimmune responses, off-target activity, and cytokine release syndromes. Some nanoplatforms and inorganic compounds need to have further development to overcome ethical issues to reach in vivo. A deeper understanding of the tumor niche is essential to discovering new pharmacological treatments and targets; thus, niches provide the optimal battlefield to win the battle against cancer.

## Figures and Tables

**Table 1:** Summary Table of the different strategies to target TME peculiarities.

Strategy	Mechanism of Action	TME Trigger	Selectivity	Development Stage	Key Challenges
pH-sensitive nanocarriers	Drug release under acidic extracellular pH	Acidic pHe (6.5–6.9)	High (TME-specific)	Preclinical/early clinical	Scale-up, EPR heterogeneity, immune response
Hypoxia-activated prodrugs	Enzymatic or chemical activation in low O_2_	Hypoxia (<2% O_2_)	Moderate–High	Preclinical/clinical	Heterogeneous oxygen levels, off-target effects
Pt(IV) complexes	Reductive activation in hypoxic/reductive areas	Hypoxia, Reductive TME	Moderate	Clinical trials (e.g., satraplatin)	Neurotoxicity, limited reduction selectivity
Gasotransmitters (NO, CO)	Redox modulation and signaling disruption	ROS, hypoxia, enzymes	Variable	Preclinical	Dosing window, systemic toxicity, targeted delivery systems

**Table 2:** Clinical studies approved for targeting or remodeling tumor microenvironment for cancer therapy. Available online: https://clinicaltrials.gov/ (accessed on 30 June 2025).

NCT Number	Study Title	Conditions	Sponsor	Study Type
NCT06790797	Investigating the Biomarkers in Tumor and Peripheral Blood to Evaluate the Efficacy of Cancer Immunotherapy in Chest Cancer Patients	Lung cancer; Esophageal cancer; In vitro stimulation of PBMCs with tumor antigen nanoparticles	Zhao Jun	Observational
NCT06776198	Precision Therapy Based on Immune Microenvironment by Transcriptome Sequencing of Osteosarcoma, a Prospective, Multi-cohort Exploratory Clinical Study	Osteosarcoma transcriptome tumor microenvironment	Peking University People’s Hospital	Observational
NCT06922825	Pilot Study Evaluating Tumor Microenvironment Interaction in Solid Tumor Patients	Solid tumor cancer;	Eben Rosenthal	Interventional
NCT02042781	Study of the Safety and Tolerability of IV Infused PG545 (Pixatimod) in Patients With Advanced Solid Tumours	Advanced solid tumours	Zucero Pty Ltd.	Interventional
NCT03844763	Targeting the Tumor Microenvironment in R/M SCCHN (Avelumab)	Head and neck cancer	Gruppo Oncologico del Nord-Ovest	Interventional
NCT04225390	Preconditioning of Tumor, Tumor Microenvironment and the Immune System to Immunotherapy (Dacarbazine)	Immunotherapy	University of Erlangen-Nürnberg Medical School	Interventional
NCT06439173	Study of the Hematopoietic Niche and the Role of Inflammation in the Pathophysiology of Cytopenias After CAR-T Cell Therapy: Potential of Therapies Directed to Repair the Bone Marrow Microenvironment	Diffuse large B cell lymphoma. Biological: CAR-T cell therapy	Instituto de Investigación Biomédica de Salamanca	Observational
NCT03647839	Modulation Of The Tumour Microenvironment Using Either Vascular Disrupting Agents or STAT3 Inhibition in Order to Synergise With PD1 Inhibition in Microsatellite Stable, Refractory Colorectal Cancer	Colorectal cancer metastatic	Australasian Gastro-Intestinal Trials Group	Interventional

## Data Availability

No new data were created or analyzed in this study. Data sharing is not applicable.

## Abbreviations

The following abbreviations are used in this manuscript:

AI	Artificial Intelligence
Akt	Protein Kinase B
ARNT	Aryl Hydrocarbon Receptor Nuclear Translocator
CORM	Carbon Monoxide-Releasing Molecules
DMSO	Dimethyl Sulfoxide
DOPE	1,2-Dioleyl-*sn*-glycrro-3-phosphoethanolamine
FGF2	Fibroblast Growth Factor 2
GOD	Glucose Oxidase
HIF	Hypoxia-Inducible Factor
Hsp90	Heat Shock Protein 90
ICAM	Intercellular Adhesion Molecule
ITH	Intratumor Heterogeneity
MAPK	Mitogen-Activated Protein Kinase
MMP	Metalloprotease
mTOR	Mechanistic Target Of Rapamycin
NORM	Nitric Oxide-Releasing Molecules
NSAID	Non-Steroidal Anti-Inflammatory Drug
PDGF	Platelet-Derived Growth Factor
PE	Dioleoyl Phosphatidylethanolamine
PEG	Polyethylene Glycol
PEI	Polyethyleneimine
PI3K	Phosphatidylinositol 3-Kinase
ROS	Reactive Oxygen Species
TGFβ	Transforming Growth Factor Beta
TME	Tumor Microenvironment
VEGF	Vascular Endothelial Growth Factor
